# Identification of subtypes of clear cell renal cell carcinoma and construction of a prognostic model based on fatty acid metabolism genes

**DOI:** 10.3389/fgene.2022.1013178

**Published:** 2022-09-16

**Authors:** Shiwen Nie, Youlong Huili, Anliang Yao, Jian Liu, Yong Wang, Lei Wang, Liguo Zhang, Shaosan Kang, Fenghong Cao

**Affiliations:** ^1^ North China University of Science and Technology, Tangshan, China; ^2^ Department of Urology, North China University of Science and Technology Affiliated Hospital, Tangshan, China

**Keywords:** fatty acid metabolic, prognostic model, tumor microenvironment, TCGA, GEO

## Abstract

**Background:** The effects of fatty acid metabolism in many tumors have been widely reported. Due to the diversity of lipid synthesis, uptake, and transformation in clear cell renal cell carcinoma (ccRCC) cells, many studies have shown that ccRCC is associated with fatty acid metabolism. The study aimed was to explore the impact of fatty acid metabolism genes on the prognosis and immunotherapy of ccRCC.

**Methods:** Two subtypes were distinguished by unsupervised clustering analysis based on the expression of 309 fatty acid metabolism genes. A prognostic model was constructed by lasso algorithm and multivariate COX regression analysis using fatty acid metabolism genes as the signatures. The tumor microenvironment between subtypes and between risk groups was further analyzed. The International Cancer Genome Consortium cohort was used for external validation of the model.

**Results:** The analysis showed that subtype B had a poorer prognosis and a higher degree of immune infiltration. The high-risk group had a poorer prognosis and higher tumor microenvironment scores. The nomogram could accurately predict patient survival.

**Conclusion:** Fatty acid metabolism may affect the prognosis and immune infiltration of patients with ccRCC. The analysis was performed to understand the potential role of fatty acid metabolism genes in the immune infiltration and prognosis of patients. These findings have implications for individualized treatment, prognosis, and immunization for patients with ccRCC.

## Introduction

Renal cell carcinoma (RCC) is one of the top 10 tumors recorded globally (Siegel et al., 2018), and according to the World Health Organization, more than 140,000 renal cell carcinoma patients die each year (Ferlay et al., 2015). The main pathological types of renal cell carcinoma include ccRCC, papillary renal cell carcinoma, and chromophobe cell renal cell carcinoma (Shuch et al., 2015; Hsieh et al., 2017), with ccRCC being the most common pathological subtype worldwide (Shen and Kaelin, 2013). Given the dangers of ccRCC, the identification of effective predictive tools and potential therapeutic targets remains of current interest.

It is well known that metabolic imbalance is one of the main characteristics of tumors, and existing scientific studies confirm that metabolic reprogramming plays a very critical role in the development of tumors (Hanahan and Weinberg, 2011; Rosario et al., 2018; Crunkhorn, 2019). Energy metabolic reprogramming, a new hallmark of tumors, enables accelerated cell growth and proliferation (Veglia et al., 2019; Corn et al., 2020). The first typical metabolic change is the Warburg effect, i.e. aerobic glycolysis. Next, there is glutamine metabolism (Wise and Thompson, 2010). Previously, studies related to abnormal fatty acid metabolism (FA) have not received much attention, but in recent years it has gradually attracted more attention as one of the features of metabolic reprogramming in tumors (Li and Zhang, 2016; Qi et al., 2019; Li et al., 2020). In many cancers, lipid uptake and storage are increased to meet cancer cell energy requirements, and fatty acids provide energy to tumor cells via β-oxidation (Cheng et al., 2018). Renal cell carcinoma has significant changes in cellular metabolism, such as FA metabolism, and RCC characterized by metabolic imbalance is considered to be a metabolic disease (Massari et al., 2015; Wettersten et al., 2017; Akhtar et al., 2018). Fatty acid synthesis is dependent on acetyl-CoA. Mutations in stearoyl-CoA desaturase 1, fatty acid synthase and acetyl-CoA carboxylase in ccRCC can lead to the substantial synthesis of acetyl-CoA, thus causing an abnormal pathway of fatty acid synthesis in ccRCC (Sajnani et al., 2017; Maan et al., 2018). Zhao et al. (2019) confirmed that multiple fatty acid metabolizing enzymes are potential prognostic markers for ccRCC, which suggests that abnormalities in fatty acid metabolism might influence the development of ccRCC. Therefore, further analysis of the impact of fatty acid metabolism-related genes (FRGs) in ccRCC may provide some reference for patient prognosis and individualized treatment.

In our analysis, we aimed to construct a prognostic signature based on TCGA (The Cancer Genome Atlas) database in conjunction with the GEO (Gene Expression Omnibus) database, using FRGs as a predictor. Patients were then distinguished into two different subtypes based on FRGs expression by unsupervised cluster analysis and, finally, the tumor microenvironment was studied in different risk groups and different subtypes. In this analysis, our results identified FRGs as a potential target for ccRCC as well as a prognostic marker. Furthermore, an attempt was made to explain the alteration of FA metabolism in the prognostic-immune-tumor microenvironment in ccRCC.

## Materials and methods

### Data collation and acquisition

To date, many sequencing data are publicly available online; therefore, we acquired the relevant data of ccRCC from TCGA (The Cancer Genome Atlas) database. There were 539 tumor samples and 79 normal samples in this dataset. Similarly, the data set GSE29609, which contains 39 tumor tissues, was obtained from the GEO (Gene Expression Omnibus) database. We also obtained clinically relevant information on the samples, and samples with incomplete clinical information were excluded from further analysis. We converted the downloaded FPKM data to TPM format. We background the adjusted and quantile the normalized TCGA data before performing the analysis. Batch effects were removed by a combat algorithm thus merging the two data sets TCGA-KIRC and GSE29609 (The log2 transform is used to normalize the data, and the combat algorithm is performed using the sva function). After excluding patients with no clinical information, a total of 569 patients were included for analysis. The independent dataset from the International Cancer Genome Consortium (ICGC) database (https://dcc.icgc.org/) was used for subsequent external validation. After excluding patients with incomplete clinical information, 90 patients were included in the ICGC cohort.

The gene collections of the KEGG fatty acid metabolic pathway, Hallmark fatty acid metabolic genes, and Reactome fatty acid metabolic genes were obtained from the Molecular Signature Database (MSigDB V7.2), and 309 FRGs were obtained after removing the overlapping parts of the three gene collections, and the specific genes are shown in Supplementary Table S1 (He et al., 2021).

**Figure:**
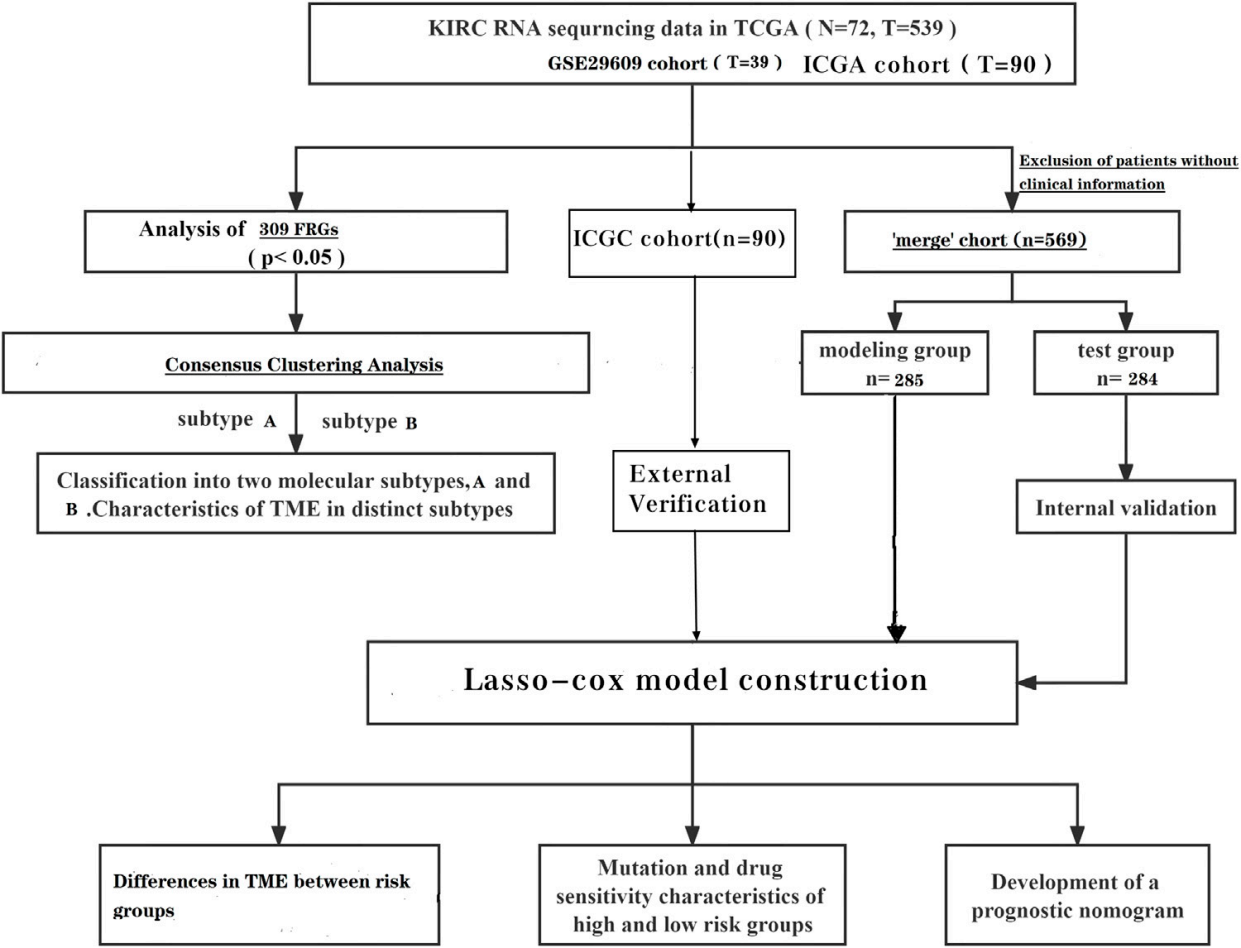


### Prognosis-related differential genes of clear cell renal cell carcinoma

Differential analysis was performed on TCGA dataset. We extracted the DEGs (differential genes) of FRGs using “limma” with a fold change of 1.5; *p* < 0.05. Further univariate cox analysis of the “merged” cohort was performed to obtain genes associated with survival time. The prognosis-related genes intersected with the differential genes of FRGs to obtain the prognosis-related DEGs (Supplementary Table S2).

### Prognostic models associated with fatty acid metabolism-related genes

The “merge” cohort was randomly assigned equally to obtain the train group and the test group. Using the train group as the base sample, the prognosis-related DEGs was analyzed using the lasso analysis and multivariate cox analysis to obtain the prognosis model.

The median FRG score in the train group can distinguish patients in the train group into low risk and high-risk groups. Survival analysis and PCA analysis were performed for the different risk groups. ROC (receiver operating characteristic) curves were plotted to verify the accuracy of the model. Finally, the correlation between clinical indicators and risk scores was assessed.

### Producing a nomogram

The nomogram was created using the “rms” package. Clinical information and risk scores are used as predictors. Each patient has a different score for different indicators. Each total score has a corresponding 1-year, 3-year and 5-year survival rate.

### Unsupervised consensus cluster analysis for fatty acid metabolism-related genes

We performed unsupervised clustering analysis with the “ConsensusClusterPlus” package. We classified the “merge” samples into different subtypes based on the expression of FRGs. To observe the differences of FRGs in different subtypes, we performed gene set variation analysis on different subtypes to further identify the differences between them. We also investigated the differences in immune infiltration between the different subtypes.

### Immune cell infiltration profile, somatic mutation profile, and drug sensitivity analysis

To further observe potential differences between different risk groups of ccRCC patients, the association between immune cells and modeled genes was studied using the CIBERSORT algorithm. Mutation data from TCGA.KIRC.varscan assessed mutations in different risk groups. The values of semi-inhibitory concentrations (IC50) of ccRCC chemotherapeutic drugs were obtained using the pRRophetic package and further observed for differences in drug sensitivity between the groups.

### Statistical

R software and Perl software were used to perform data analysis (“*” = *p*< 0.05; “**” = *p*< 0.01; “***” = *p*< 0.001). Kaplan-Meier analysis and log-rank test were used to compare OS between subgroups. Immune cell infiltration and TME scores were compared between high- and low-risk groups and between subtypes using the Wilcoxon test. Spearman correlation analysis was used to compare correlations between the degree of immune cell infiltration and risk scores.

## Results

### Construction and validation of prognostic models

**FIGURE 1:**
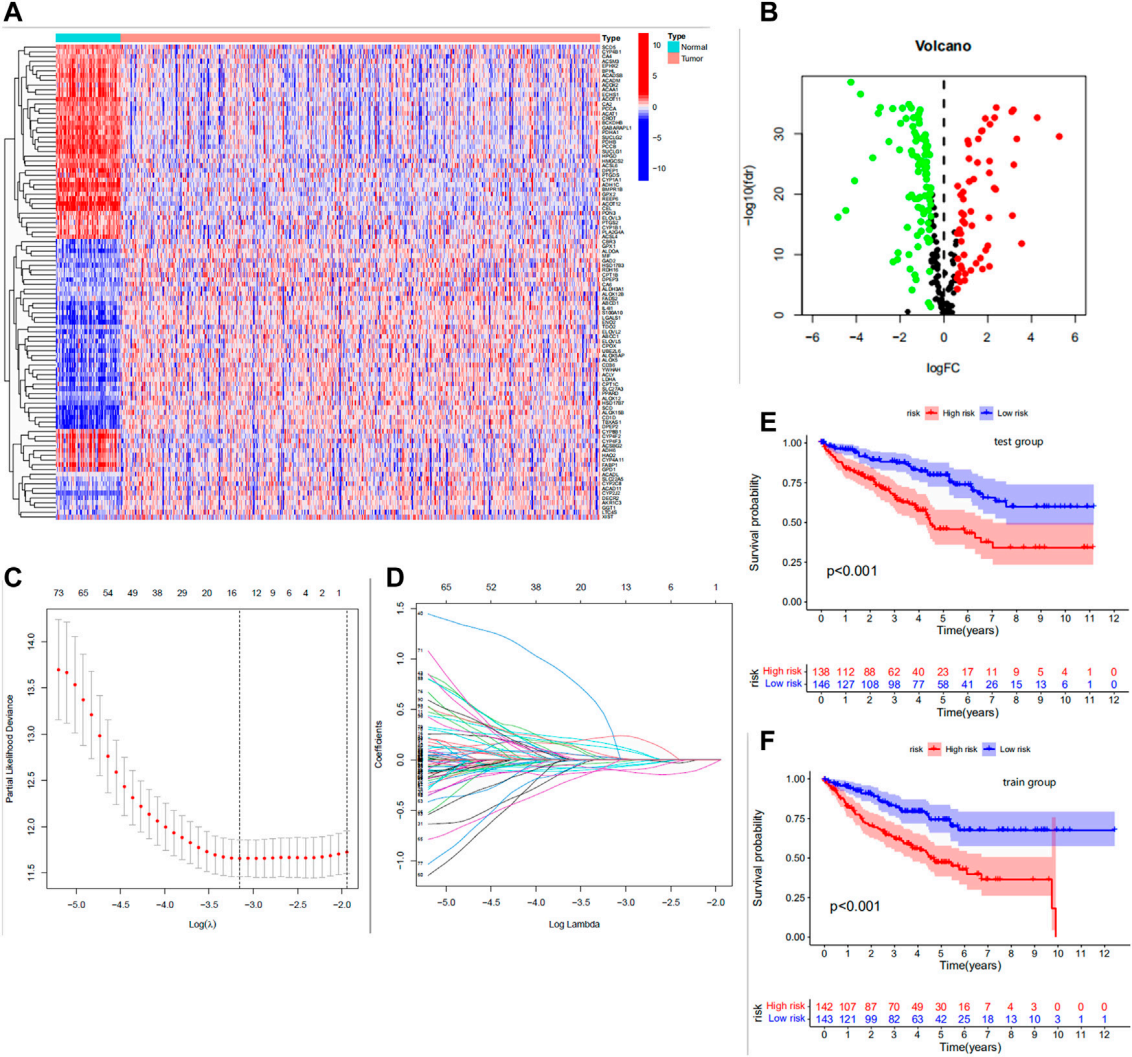
**(A)** Heat map of differential expression of the top 100 most significantly differentially expressed fatty acid metabolism genes. Red for high expression, blue for low expression. **(B)** Volcano plot of 151 differentially expressed genes. Red dots indicate upregulated genes, black dots indicate insignificant differences, and green dots indicate downregulated genes. **(C)** Cross-validation was performed to select the minimum lambda value to obtain the modeled genes. **(D)** LASSO coefficient profiles of the 99 FRGS. **(E,F)** The Kaplan-Meier analysis in the test/train group.

We extracted DEGS of 151 fatty acid metabolism-related genes using “limma,” of which 94 were down-regulated and 57 were up-regulated (Figures 1A,B). Univariate cox analysis was used to obtain genes associated with survival time. The intersection of prognosis-related genes with the differential genes of FRGs was taken to obtain 99 prognosis-related DEGs (Supplementary Table S2). The samples were equally divided into the train group (*n* = 285) and the test group (*n* = 284). Based on the 99 prognosis-related DEGs, we performed LASSO regression (Figures 1C,D) and multivariate cox analysis to obtain a prognostic model including 11 predictors (HSD17B3, HPGD, CEL, PTGDS, SCD5, DPEP1, GAD2, ADH6, ALOX12B, IL4I1, and ENO2). This model can be expressed by the formula:

Risk score = (0.2468 * HSD17B3) + (−0.1651 * HPGD) + (0.3335 * CEL) + (0.1054 * PTGDS) + (−0.1180 * SCD5) + (−0.1239 * DPEP1) + (1.2038 * GAD2) + (−0.1880 * ADH6) + (−0.5212 * ALOX12B) + (0.2610 * IL4I1) + (0.1566 * ENO2)

**FIGURE 2:**
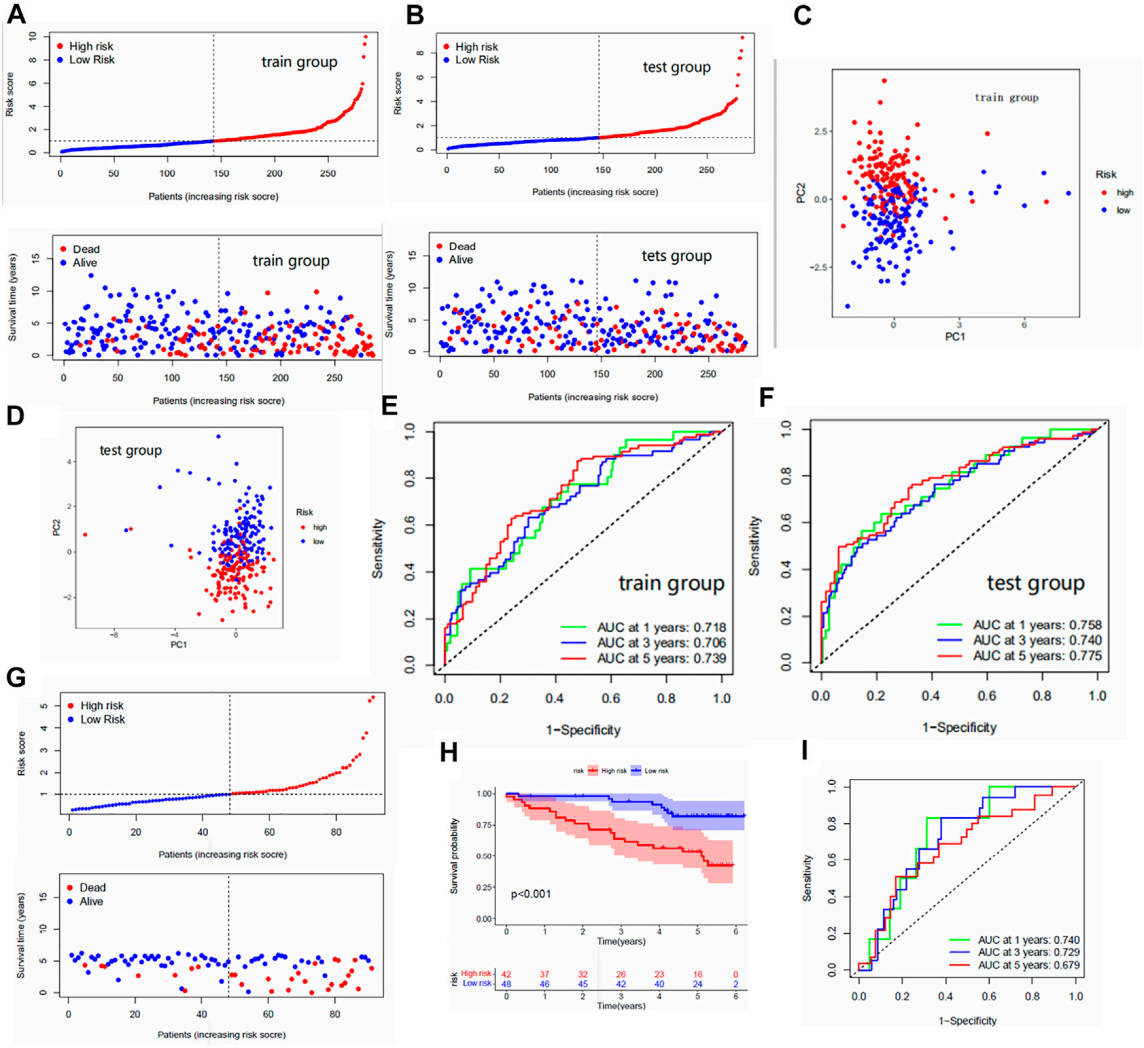
**(A,B)** Risk curves for the train and test groups. These include scatter plots showing risk scores and patient survival status, and ranked plots in order of increasing risk scores. **(C,D)** PCA results for the train/test groups. Each dot represents a patient. **(E,F)** The area under the ROC curve for the train/test group. The area under the ROC curve represents the accuracy of the model in predicting patient survival, where the larger the area, the higher the accuracy. **(G)** Risk curves for the ICGC cohort. **(H)** The Kaplan-Meier analysis in the ICGC cohort. **(I)** The area under the ROC curve for the ICGC cohort.

The median FRG score of the train group distinguished patients in the train group into high and low risk groups. Similarly, the test group was also distinguished into two different risk groups. The Kaplan-Meier analysis are shown in Figures 1E,F; we observed significantly lower os (overall survival) in patients with high risk scores than in patients with low risk scores, which laterally reflects the reliability of the risk scores. The risk curve results show a gradual increase in the number of high-risk patients with increasing risk scores and a higher number of deaths in patients with high risk scores (Figures 2A,B). The PCA analysis showed us the excellent discriminatory ability of the model (Figures 2C,D). The area under the ROC curve was greater than 0.7 for both the train and test groups (Figures 2E,F). This can indicate the high accuracy of the model prediction ability. To further determine the predictive power of the model, we externally validated the model using the ICGC cohort. The risk curve and survival curve also demonstrated the superior performance of the model (Figures 2G,H). We found that the ROC curves at 1, 3, and 5 years exceeded 0.65 (Figure 2I).

### Independent prognostic analysis

**FIGURE 3:**
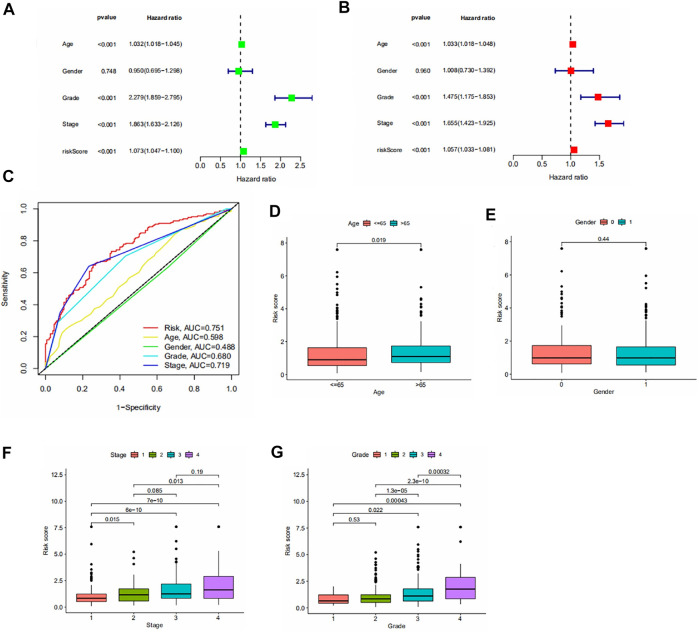
**(A,B)** Forest plot of univariate/multivariate independent prognostic analysis (*p* < 0.05 is statistically significant, the larger the hazard ratio, the higher the correlation). **(C)** Area under the roc curve for risk scores and clinical information. **(D–G)** Results of the clinical correlation analysis. Differences in risk scores by age, gender, stage, and grade.

We performed the independent prognostic analysis of clinical indicators and risk scores. We found that age, grade, stage, and risk score could be used as prognostic factors independently of other factors (Figures 3A,B). As shown in Figure 3C, we observed that the risk score had the largest area under the roc curve, which implies that the risk score was more accurate than age, grade, and stage.

To assess the association between risk scores and clinical parameters, we performed a clinical correlation analysis. The findings are shown in Figures 3D–G. It can be seen that age and gender have no significant effect on the risk score. The risk scores increased with increasing grade and stage levels.

### A new nomogram

**FIGURE 4:**
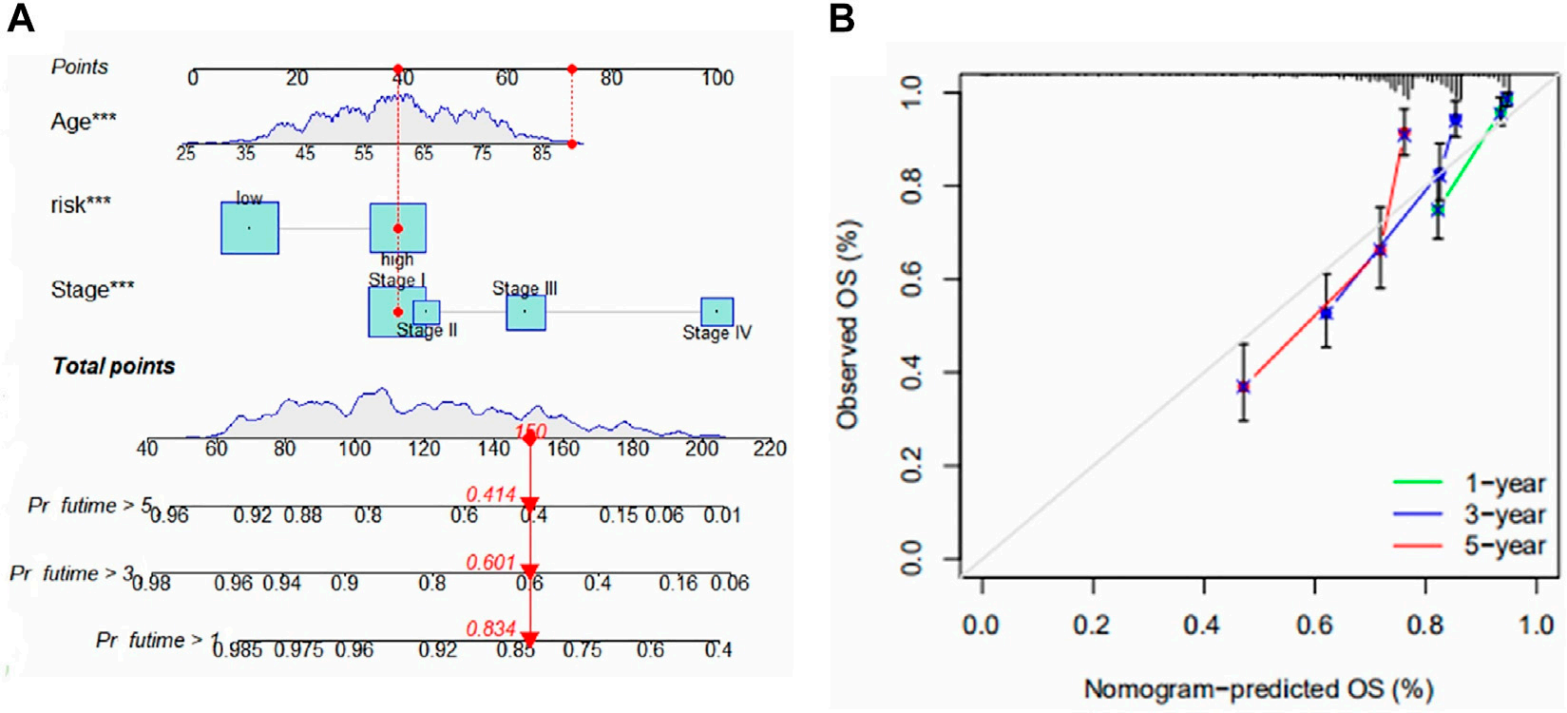
**(A)** Nomogram for predicting 1-, 3-, and 5-year survival for ccRCC. The figure shows the predicted results for the 17th patient in the train group. This patient belongs to the high-risk group of patients, age 90, and at stage I. The scores of each index were 40, 70, and 40, total scores of 150, 1-year, 3-year, and 5-year survival rates were 0.834, 0.601 and 0.414. **(B)** Calibration chart for the evaluation of nomogram accuracy.

To be able to use this model in a clinical setting, we used risk score, stage, and age as predictors thus constructing a Nomogram (Figure 4A). The results of the calibration plots showed a good predictive effect of the nomogram (Figure 4B).

### Identification of fatty acid metabolism subtypes in clear cell renal cell carcinoma

**FIGURE 5:**
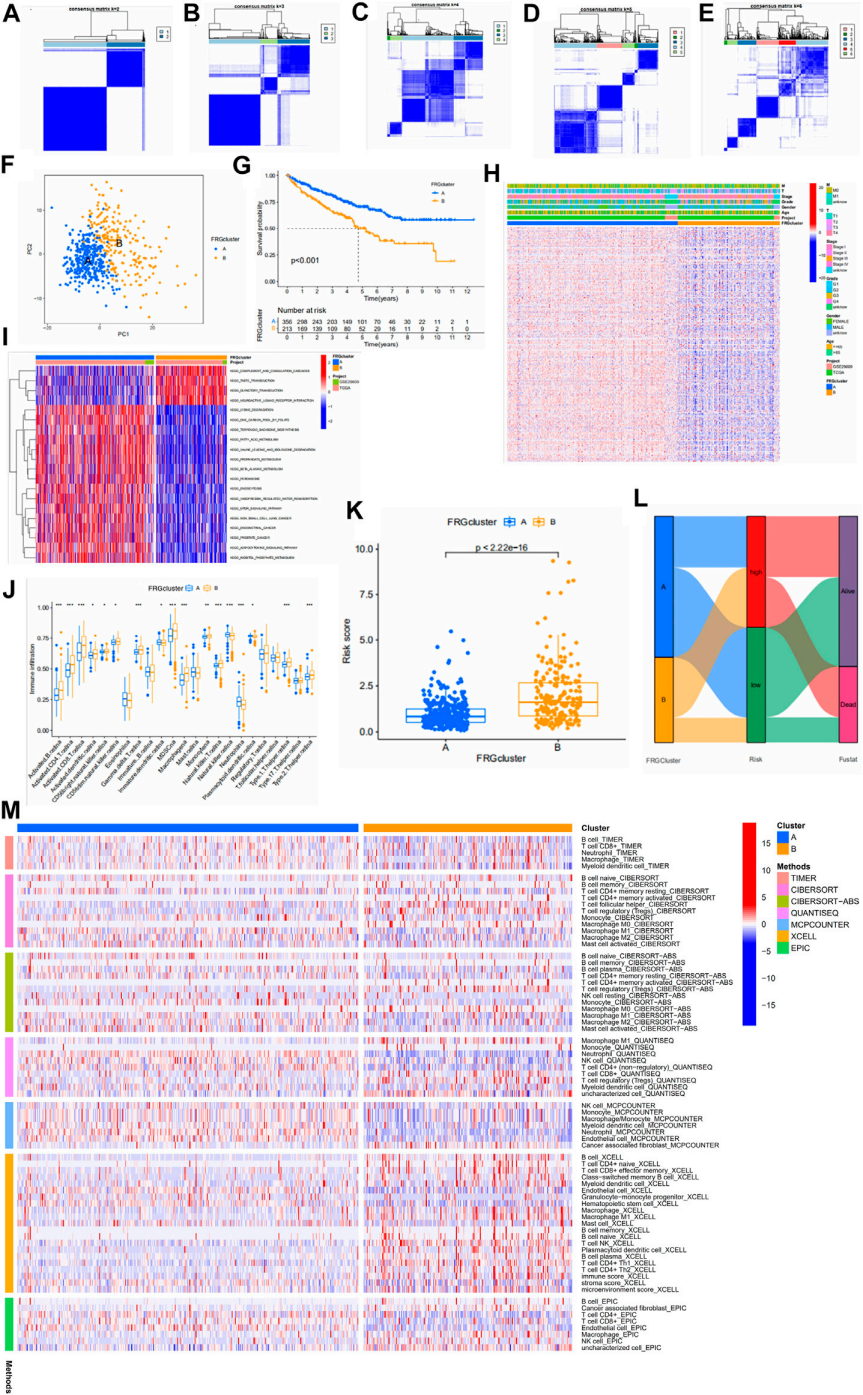
**(A–E)** Results of unsupervised consensus clustering analysis. Defined as two, three, four, five, and six subtypes and their areas. **(F)** The results of PCA analysis between the two subtypes. The figure shows significant differences in fatty acid metabolism-related genes between the two subtypes. **(G)** Survival curves between the two subtypes. **(H)** Comparison between clinical indicators and the two subtypes. **(I)** Differences in biological pathways analyzed by GSVA (gene set variation analysis) in two subtypes. Red for pathways of activation and blue for pathways of inhibition. **(J)** Differences in the level of immune cell infiltration in the two subtypes. **(K)** Association of different subtypes with risk scores. Each point represents a patient. **(L)** Sankey diagram showing the distribution of patients. **(M)** Multiple methods to calculate the immune cell content of two fatty acid metabolic subtypes.

To understand the expression pattern of fatty acid metabolism in ccRCC, all samples were classified into different subtypes using cluster analysis based on the expression of 309 FRGs. k = 2 was appropriate as seen in Figures 5A–E. Therefore, we classified the patients into subtypes A and B. PCA analysis showed a significant difference in FRGs expression between subtypes A and B (Figure 5F). Kaplan-Meier analysis showed higher os in subtype A than in subtype B (Figure 5G). The comparison of clinical indicators between the two subtypes is shown in Figure 5H, which shows significant differences in FRGs expression and clinical information between the two subtypes. GSVA enrichment analysis showed enrichment in multiple cancer pathways in subtype A, including non-small cell lung cancer, endometrial cancer, and prostate cancer. Tumor signaling pathways were also significantly enriched in subtype A (Figure 5I). The difference in immune cell content between the two subtypes was calculated using the CIBERSORT algorithm. In tcga cohort, we observed that 17 of the 23 immune cells differed significantly between subtypes A and B, and the majority of immune cells were more infiltrated in subtype B than in subtype A (Figure 5J). Some immune cells show a higher degree of infiltration in A than in B. This might be since these immune cells have different roles from other immune cells in both subtypes. In addition, there are significant differences in the risk scores of the different clusters, and it can be seen that the cluster A has a significantly lower risk score than B (Figure 5K). Furthermore, the majority of patients in cluster A belonged to the low risk group and the majority of patients in cluster B belonged to the high-risk group (Figure 5L). Finally, we used multiple platforms to verify the immune infiltration between fatty acid metabolism subtypes. We found an enrichment of immune cells in subtype B, which further suggests a higher degree of immune infiltration in subtype B (Figure 5M).

### Risk scores and tumor microenvironment

**FIGURE 6:**
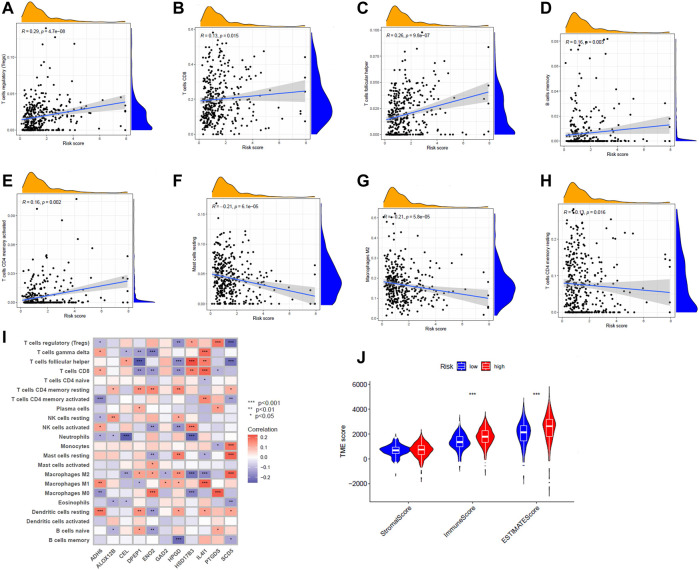
**(A–H)** Correlation between risk scores and various immune cells. **(I)** Correlation of 11 genes and immune cell content. Red means positive association, and purple means negative association. The shade of the color indicates the strength of the correlation. **(J)** Violin plot indicating differences in tumor microenvironment scoring for different risk groups.

The CIBERSORT algorithm was further used to explore the relationship between risk score and immune cells. In tcga cohort, we found that the risk score was positively correlated with Tregs, CD8^+^ T cells,T follicular helper cells, memory B cells, and activated memory CD4^+^ T cells (Figures 6A–E). Resting mast cells, M2 macrophages, and resting memory CD4^+^ T cells were negatively correlated with the risk score (Figures 6F–H). We observed the connection between the 11 genes that constitute the model and immune cell infiltration. These genes could be found to be significantly associated with immune cells (Figure 6I). There were also significant differences in immune scores between risk groups (Figure 6J). In addition, we show the protein expression of model genes in normal and tumor tissues (Supplementary Figure S8). These results were obtained from the HPA (Human Protein Atlas, https://www.proteinatlas.org/) database.

### Mutation status and drug sensitivity analysis

**FIGURE 7:**
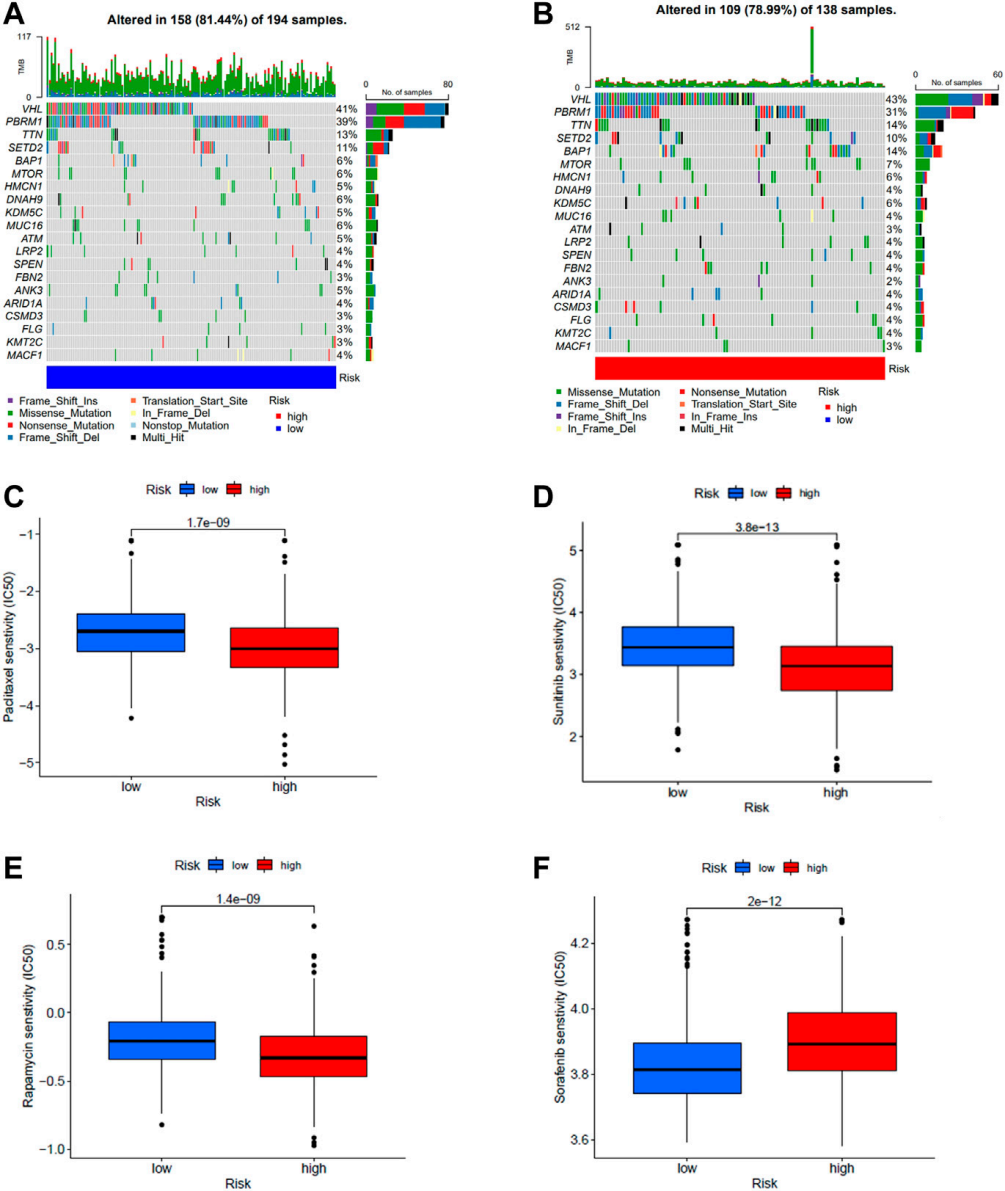
**(A,B)** Waterfall plots of somatic mutation characteristics were created for high and low risk groups. Each column represents a patient. **(C–F)** Differences in drug sensitivity of paclitaxel, sunitinib, rapamycin, and sorafenib between high- and low-risk groups.

The mutation data from TCGA. KIRC.varscan revealed the same top twenty mutated genes between the two groups and a higher number of mutated samples in the low-risk group than in the high-risk group (Figures 7A,B). To observe the differences in drug sensitivity of commonly used chemotherapeutic agents between the different risk groups, using the “pRRophetic” package for drug sensitivity analysis, we observed that patients in the high-risk group were more sensitive to paclitaxel, sunitinib, and rapamycin, while patients in the low-risk group were more sensitive to sorafenib (Figures 7C–F). Subtype B were more sensitive to Paclitaxel, sunitinib, and rapamycin (Supplementary Figure S9).

## Discussion

In the current situation, the mortality rate of ccRCC has still not decreased. As a tumor with metabolic disease nature, the effect of fatty acid metabolism on ccRCC is unknown. To understand the impact of fatty acid metabolic patterns, we distinguished two subtypes with different TME and prognosis by the expression of FRGs and identified the gene signature associated with the prognosis of ccRCC.

Some research tables have demonstrated that the TME has a significant impact on the development of tumors (Hinshaw and Shevde, 2019). The immune cell component of the TME contains lymphocytes, granulocytes, and macrophages. These immune cells play different roles in various immune responses that promote or inhibit tumor survival (Seager et al., 2017). Previous studies have found that CD4^+^ T cells have a positive impact on tumor immunity (Saito et al., 2016). Macrophages, on the other hand, have a complex role (Dehne et al., 2017). Among them, M1 macrophages have an anti-tumor immune role and M2 suppress tumor immunity and promote tumor growth (Sica et al., 2006; Jeannin et al., 2018; Chen et al., 2019). Fatty acids influence the function of immune cells in TME (Tanaka and Sakaguchi, 2017), so it is worth exploring the differences in the degree of immune cell infiltration by different subtypes in our analysis of fatty acid metabolism genes. Some immune cells with antitumor properties can be seen to be enriched in subtype B, yet the Kaplan-Meier analysis of subtype B demonstrated a poor prognosis. We speculate that the effect of fatty acids on immune cell function produced these results. Tanaka and Sakaguchi (2017) found that Tregs infiltration was associated with a poor prognosis, and the amount of Tregs in our analysis was positively correlated with the risk score, which implies that the higher the infiltration of Tregs, the higher the risk, which is consistent with previous reports. We found from the analysis that different subtypes represent different prognoses. Thus, subtype A can try to predict a good prognosis for ccRCC patients. Likewise, subtype B can predict a poor prognosis in patients. The different levels of immune cell infiltration in the two subtypes may also cause patients to show different outcomes when receiving immunotherapy.

We constructed a prognostic model based on fatty acid metabolism-related genes, which included 11 fatty acid metabolism genes (HSD17B3, HPGD, CEL, PTGDS, SCD5, DPEP1, GAD2, ADH6, ALOX12B, IL4I1, ENO2). Some of these 11 fatty acid metabolism genes have been previously reported, and the results of Song et al.'s analysis of selective splicing signals of more than 10,000 genes in ccRCC demonstrated that SCD5 is one of the potential markers of tumor prognosis (Song et al., 2019). Several reports assessed the expression levels of DPEP1 in various tumor cells and revealed opposite patterns depending on the tumor type. For example, DPEP1 expression deficiency was associated with breast cancer and Wilms’s tumor (Austruy et al., 1993; Green et al., 2009). Antwi et al. (2018) confirmed that mutations in ADH6 are closely associated with the risk of renal cell carcinoma. ALOX12B has been reported to be associated with a variety of cancers, including renal cell carcinoma, lung cancer, breast cancer, and vulvar epidermoid carcinoma (Agarwal et al., 2009; Lee et al., 2009; Shen et al., 2009; Rooney et al., 2015). ALOX12B inhibits immune cytolytic activity in breast and renal cell carcinoma (Rooney et al., 2015), and inhibition of ALOX12B reduces the proliferation of vulvar epidermoid carcinoma cells (Agarwal et al., 2009). IL4I1 is a fatty acid metabolism-related immune checkpoint that activates AHR and accelerates tumor growth (Sadik et al., 2020). The above genes have already been reported and the currently unreported genes could provide leads for further related studies. In addition, compared to previous work, Chen et al. (2021) performed a series of analyses based on metabolic genes in ccRCC, but metabolic alterations encompass multiple pathways. We performed studies in individual pathways (fatty acid metabolic pathway). Wei et al. (2022) constructed a prognostic model based on fatty acid metabolic genes, and the prognostic model we constructed and the identified fatty acid metabolic subtypes can be complementary to it.

The present research has some limitations and a large sample of ccRCC patients is needed to validate the accuracy of the model and the stability of the stratification. Furthermore, *in vivo* or *in vitro* experimental validation would better and more fully confirm the results of our analysis. In addition, treated patients may affect the expression of FRGs, which may have some impact on the results of our analysis.

## Conclusion

In summary, we obtained two subtypes of ccRCC associated with fatty acid metabolism. The two subtypes represent two different prognoses and have different immune infiltration outcomes. We developed 11 FRGs as prognostic models for the signature and established a nomogram to demonstrate the specific prognosis more precisely. These have implications for the individualized treatment, prognosis, and tumor microenvironment of ccRCC patients.

## Data Availability

The original contributions presented in the study are included in the article/Supplementary Material, further inquiries can be directed to the corresponding author.

## Abbreviations

FRGs, fatty acid metabolism-related genes; DEGs, Differentially expressed genes; TCGA, The Cancer Genome Atlas; ROC, Receiver operating characteristic; LASSO, least absolute shrinkage and selection operator; PCA, principal component analysis; GSVA, Gene set variation analysis; OS, Overall survival; TME, Tumor microenvironment; ccRCC, clear cell renal cell carcinoma; GEO, Gene Expression Omnibus; ICGC, International Cancer Genome Consortium.
