# Supplementary figures and images for: Identification of subtypes of clear cell renal cell carcinoma and construction of a prognostic model based on fatty acid metabolism genes
Source: Front Genet. 2022 Sep 16;13:1013178. doi: 10.3389/fgene.2022.1013178 (PMC9523225; doi:10.3389/fgene.2022.1013178)

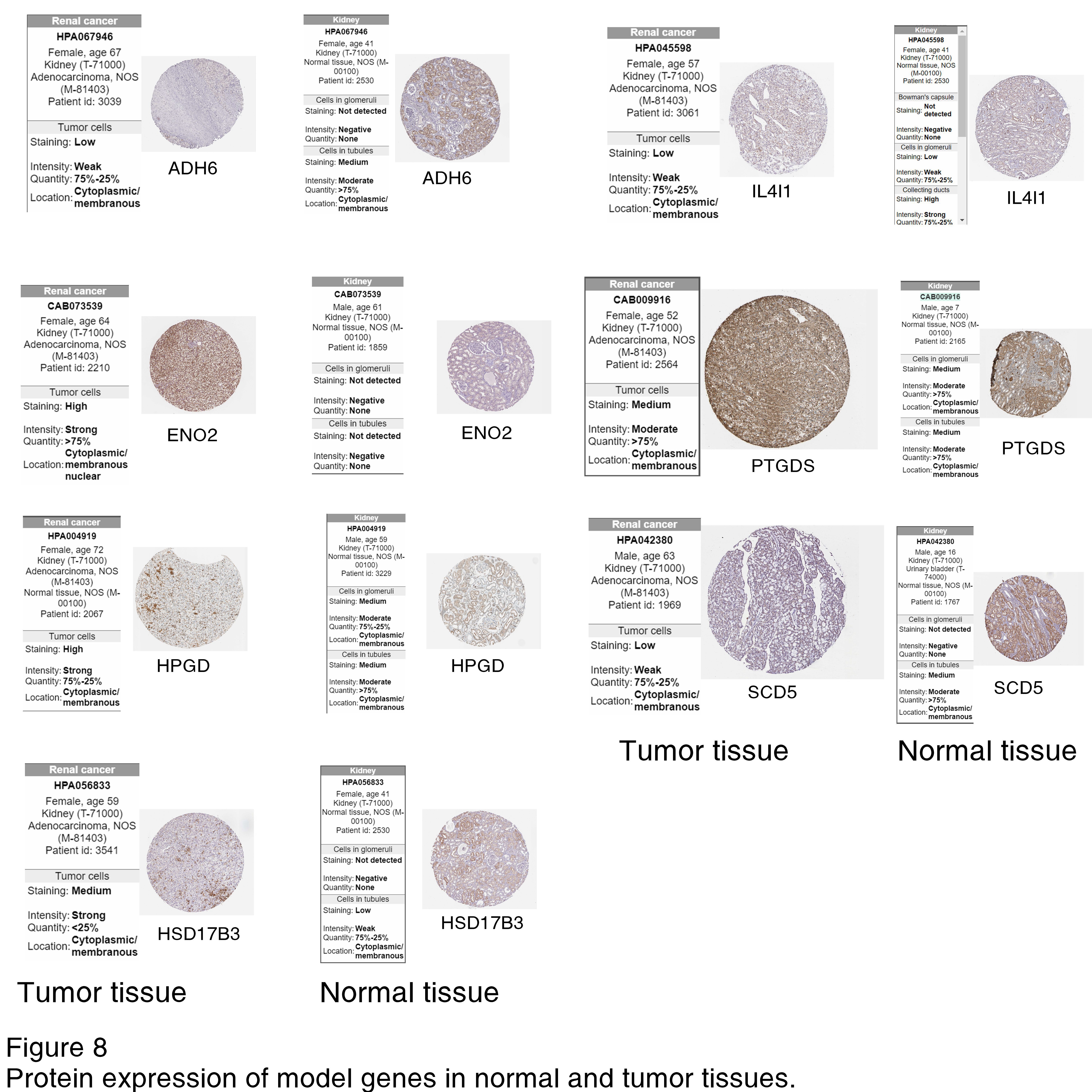

Supplement: Supplementary file 1 [file Image1.JPEG]

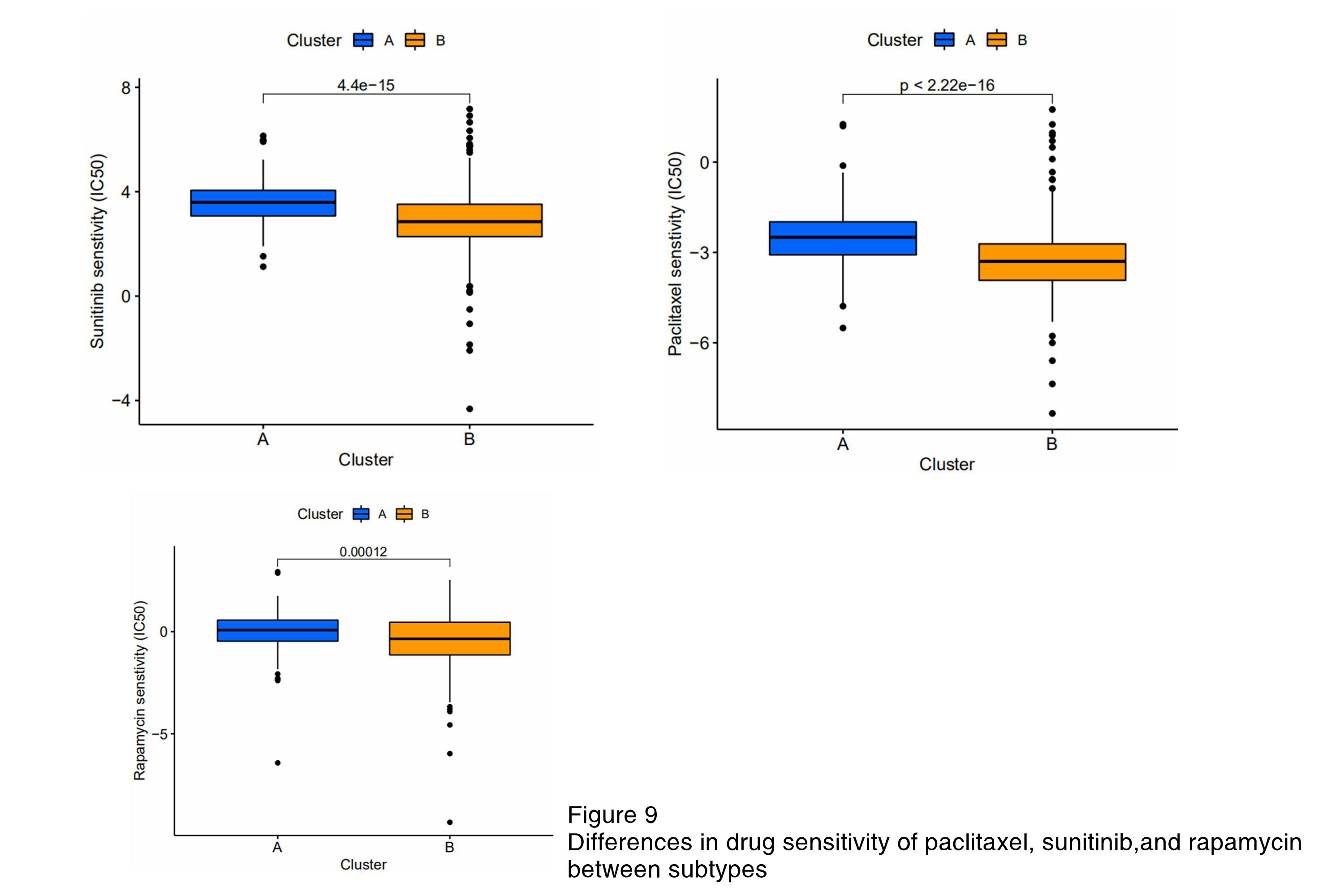

Supplement: Supplementary file 2 [file Image2.JPEG]
